# Pathogenic role of the vitreous in angle-closure glaucoma with autosomal recessive bestrophinopathy: a case report

**DOI:** 10.1186/s12886-020-01543-5

**Published:** 2020-07-09

**Authors:** Yan Shi, Jiaxin Tian, Ying Han, Julius Oatts, Ningli Wang

**Affiliations:** 1 Beijing Tongren Eye Center, Beijing Tongren Hospital, Beijing Institute of Ophthalmology, Capital Medical University, Beijing, 100730 China; 2 Department of Ophthalmology, University of California, San Francisco School of Medicine, San Francisco, CA USA; 3 Beijing Tongren Hospital, 1 Dongjiaominxiang Street, Dongcheng District, Beijing, 100730 China

**Keywords:** Autosomal recessive bestrophinopathy, Angle closure glaucoma, Small dose transscleral cyclophotocoagulation, Vitreous liquefaction

## Abstract

**Background:**

Autosomal recessive bestrophinopathy (ARB) is caused by homozygous or compound heterozygous mutations in the *BEST1* gene and always accompanied with refractory angle-closure glaucoma (ACG). The exact mechanism for the pan-ocular abnormalities in ARB is still unknown and the management of ACG in these cases is challenging.

**Case presentation:**

A 26-year-old patient with a novel autosomal–recessively inherited c.1 A > G variant in *BEST1* diagnosed as ARB and ACG, presented as widespread vitelliform deposits in the posterior pole, retinoschisis in the macula, vitreoretinal interface abnormalities, shallow anterior chamber depth (ACD) and angle closure with uncontrolled intraocular pressure (IOP). Combined phacoemulsification, intraocular lens implantation and goniosynechialysis did not improve any clinical presentation. However, low dose transscleral cyclophotocoagulation with subsequent vitreous liquefaction effectively lowered IOP, deepened ACD, and rehabilitated retinoschisis in both eyes.

**Conclusions:**

This case implied vitreous condition may play a pathogenic role in formation of retinoschisis and ACG in the patients with ARB. Treatments that induce vitreous liquefaction could be used to treat young ACG patients with ARB or other kinds of ACG to avoid vision-threatening post-operative complications.

## Background

Autosomal recessive bestrophinopathy (ARB) is caused by homozygous or compound heterozygous mutations in the BEST1 gene. The clinical features of ARB include widespread retinal pigment epithelium (RPE) irregularities, vitelliform deposits in the posterior pole, and the presence of intraretinal and subretinal fluid in the macula [1]. Angle-closure glaucoma (ACG) has also been described in the setting of ARB, possibly secondary to anterior segment dysgenesis [2]. Previous studies have shown that younger ACG patients, especially with ARB, develop malignant glaucoma more frequently than older ACG patients after trabeculectomy [3]. The early onset age, the refractory to IOP control and the high incidence of surgical complications in these ACG patients challenge glaucoma surgeons. Here we reported a ARB case with a novel variant, and firstly introduced a successful and simple treatment regimen to manage ACG and retinoschisis in this patient.

## Case presentation

A 26-year-old woman who had given birth 3 months prior presented with progressive bilateral blurred vision and ocular pain for the preceding 10 months. She initially presented to a local hospital where her best corrected visual acuity (BCVA) was 20/2000 and 20/400 in the right and left eye respectively, and intraocular pressure (IOP) was 50 mmHg in each eye. She was prescribed brinzolamide, latanoprost, brimonidine tartrate, and carteolol eye drops; however, her IOP remained in the 30’s and her vision continued to decline. On further questioning, she did recall that her vision was not good from childhood, and had noted a recent change. There was no history of eye diseases in the family, no history of ocular trauma or systemic diseases, and her recent pregnancy and delivery were both uncomplicated.

**Fig. 1:**
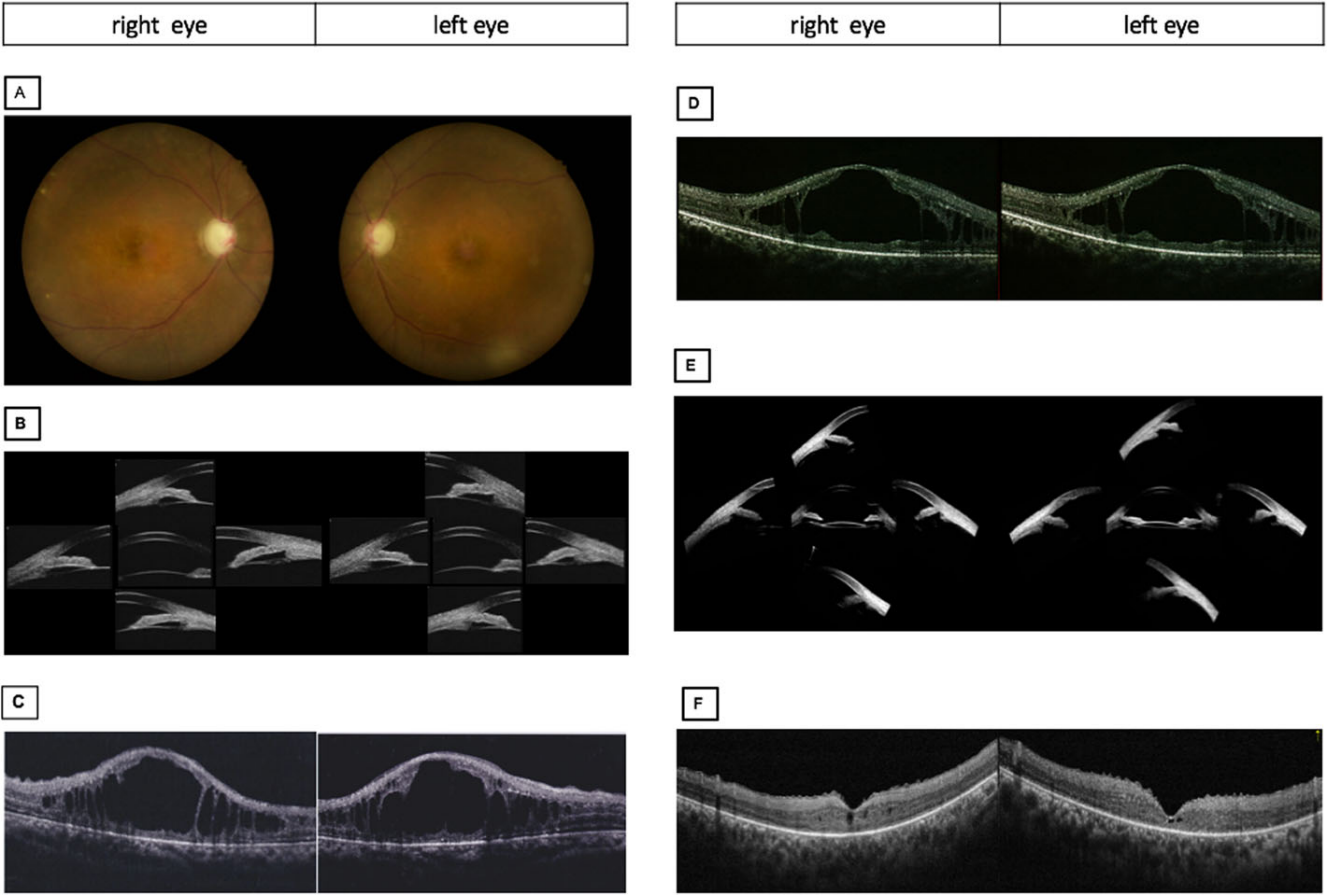
Pre-operative and post-operative ocular imaging of the patient. **a** Pre-operative fundus photographs of both eyes revealing 0.95 cup-to-disc ratio, macular pucker with mild vascular tortuosity and discrete, round, yellow-white deposits scattered throughout the macula and posterior pole. Pre-operative ultrasound biomicroscope (UBM) images showing angle closure, iridoncosis, anteriorly rotated iris root and ciliary body in both eyes. Pre-operative spectral domain optical coherence tomography (SD-OCT) showing epiretinal membranes and retinoschisis. **d** SD-OCT following phacoemulsification with intraocular implantation of lens and goniosynechialysis showing largely unchanged findings. **e** UBM of both eye showed the increased space between iris and ciliary body after small dose transscleral cyclophotocoagulation. **f** SD-OCT following small dose transscleral cyclophotocoagulation showing complete resolution of retinoschisis

On presentation, her BCVA was count fingers at 10 cm in the right eye and 20/600 in the left eye. IOP measured by Goldman applanation tonometry was 25 mmHg in the right eye and 31 mmHg in the left eye. Positive findings on slit-lamp examination of both eyes revealed deep anterior chamber centrally but shallow peripherally without inflammation or posterior synechiae. The lenses were clear. Fundus examination was notable for bilateral cup-to-disc ratio of 0.95, horizontal macular pucker with mild vascular tortuosity, and discrete, round, yellow-white deposits of variable sizes scattered throughout the macula and posterior pole (Fig. 1a). Static gonioscopy demonstrated circumferential grade IV angle by Scheie grading [4] with 2–3 clock hours open in each eye on dynamic gonioscopy. Ultrasound biomicroscopy (UBM) confirmed angle closure in the setting of iris thickening and plateau iris-like configuration with an anteriorly rotated ciliary body and iris root (Fig. 1b). IOL Master measured anterior chamber depth (ACD) of 2.64 mm in right eye and 2.77 mm in left eye and axial length of 21.88 mm in each eye. Spectral domain optical coherence tomography (SD-OCT) revealed cystoid macular edema associated with retinoschisis as well as increased reflectivity of the vitreoretinal interface in both eyes with pre-retinal tractional membranes (Fig. 1c). Subfoveal choroid thickness measured 401 μm in both eyes, thicker than the previously reported normal range in the Chinese population [5] .

**Fig. 2:**
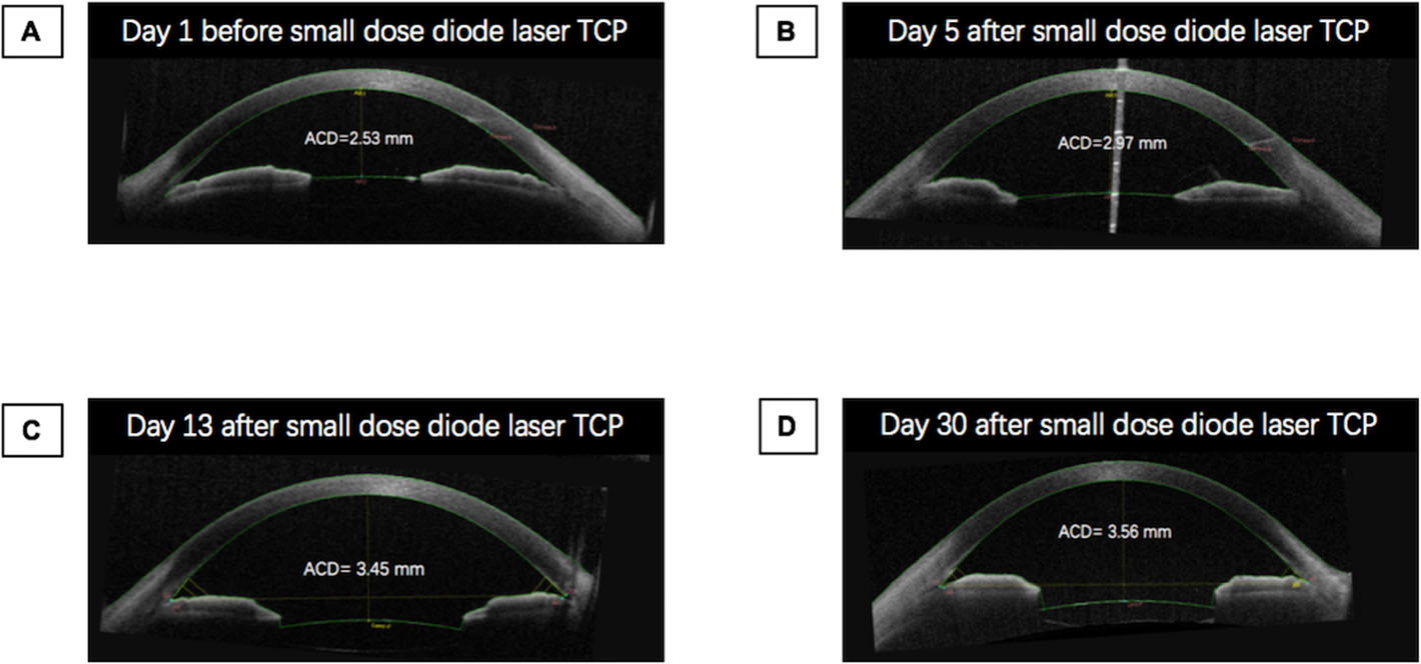
Change in anterior chamber depth in right eye during treatment. The anterior chamber depth (ACD) was measured by anterior segment optical coherence tomography. TCP: Transscleral cyclophotocoagulation

Given the elevated IOP on maximum medical therapy with angle closure and extensive peripheral anterior synechiae, the decision was made to proceed with clear lens extraction with intraocular lens implantation and goniosynechialysis in the left eye with a higher IOP. On post-operative day 1, the visual acuity was 20/800 with an IOP of 44 mmHg and unchanged ACD. Malignant glaucoma was suspected and Nd:YAG laser capsulotomy was performed in an attempt to balance the pressure between the vitreous cavity and anterior chamber, but this did not result in any improvement. Two weeks post-operatively with no improvement in the patient’s condition, diode laser transscleral cyclophotocoagulation (TCP) was performed using 10 shots over 180-degrees with power of 1800 mW and 2 s duration (OcuLight SLx 810 nm diode laser and G-probe, Iris Medical, Mountain View, CA). One day following the laser, IOP in the left eye decreased to 25 mmHg with BCVA improving to 20/667 and slightly deepened ACD without anti-glaucoma medications. By post-operative day 4, the IOP had decreased to 15 mmHg with significant improvement in ACD to 3.014 mm. Concurrently, anterior vitreous liquefaction with increased vitreous motility were noted on slit-lamp examination. By post-operative week 5, the IOP remained normal with further deepened ACD to 3.427 mm. UBM showed increased space between the ciliary body and iris root with a moderate backward rotation of the ciliary body. Interestingly, following TCP, the retinoschisis in the left eye resolved completely but with no improvement after lens extraction with intraocular lens implantation and goniosynechialysis (Fig. 1d). One week following TCP on the left eye, the right eye underwent combined phacoemulsification, intraocular lens implantation, and goniosynechialysis followed by TCP 1 week later. The right eye had a similar course of IOP control, anterior chamber deepening, backward ciliary body rotation, and resolution of the retinoschisis (Fig. 1e, f, Fig. 2). No complication occurred after TCP.

**Fig. 3:**
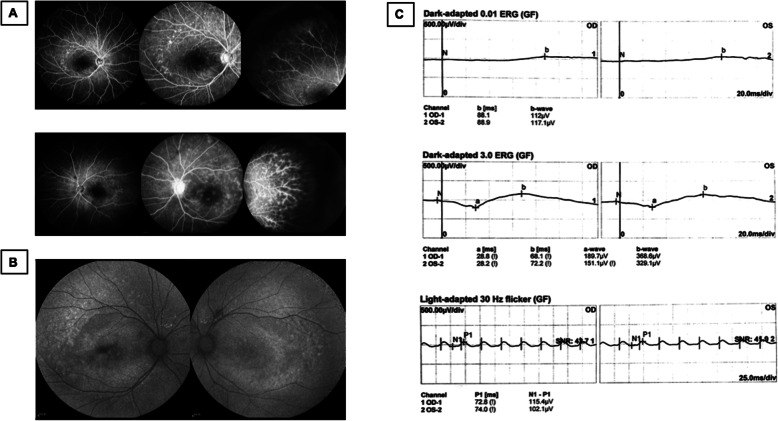
Ophthalmologic examination for final diagnosis. **a** Fundus fluorescein angiography of both eyes revealed scattered hyper-fluorescence existed in the posterior pole on early stage and didn’t change until late stage. Capillary leakage was obvious in peripheral retina in late stage. The timing of the right eye was at 0:45.97, 2:41.00 and 4:11.62 respectively. The timing of the left eye was at 0:23.23, 1:51.67 and 10:21.34 respectively. **b** Fundus autofluorescense of both eyes demonstrated hyper-autofluorescence in the posterior poles was in accord with fundus fluorescein angiography. Some hypo-autofluorescence also could be seen in the macula. **c** Flash electroretinogram showed a decreased rod photoreceptor maximal reaction amplitude with no obvious change of cone photoreceptor reaction amplitude in 30 Hz flicker

**Fig. 4:**
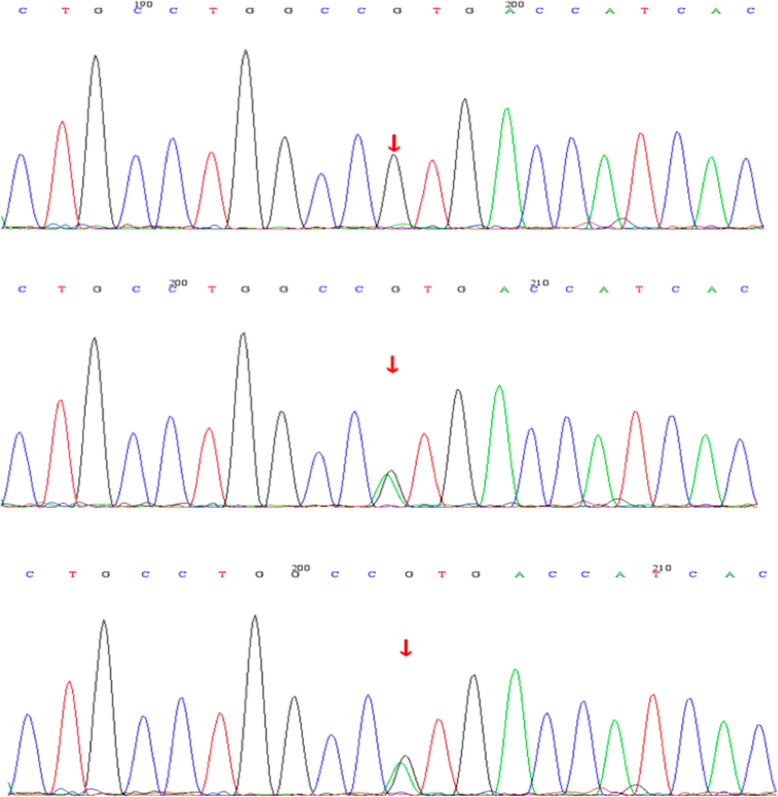
Bidirectional Sanger sequencing of the patient and her parents. The upper one showed a homozygotic mutation, c.1 A > G, p.M1V in the patient. The middle and inferior sequences showing a heterozygous mutation, c.1 A > G were shared by her parents

The patient underwent fundus fluorescein angiography 10 days following TCP on the right eye and 24 days following TCP on the left eye. This showed scattered early hyperfluorescence in the mid-periphery around the arcades with no significant change in late stages. The peripheral retina demonstrated capillary leakage in late stage images (Fig. 3a). Fundus autofluorescence showed perifoveal hypo-autofluorescence in the right worse than left eyes and hyper-autofluorescence in the posterior pole corresponding to the areas of hyperfluorescence seen on fundus fluorescein angiography (Fig. 2b). An extensive lab workup for inflammatory causes of macular edema with peripheral vascular leakage was negative, including: antinuclear antibodies, anti-double-stranded DNA antibodies, anti-neutrophil cytoplasmic antibodies, human leukocyte antigen -B27, interferon gamma release assay, serum cortisol, erythrocyte sedimentation, anti-guanidine amino acid peptide antibodies, anti-cardiolipin antibodies, anti-β2-glycoproteinIantibodies, and rheumatoid factor within normal range. Electrooculography could not provide a reliable Arden index due to the inability to obtain a basic potential in the setting of the patient’s poor vision. Flash electroretinogram showed a decreased rod photoreceptor maximal reaction amplitude. The reaction amplitude of cone photoreceptor in 30 Hz flicker had no obvious change (Fig. 3c). Based on the fundus findings and the results of ancillary testing, a diagnosis of ARB was made. Whole-exome sequencing of this patient identified a novel homozygous missense mutation, c.1 A > G, p.M1V in BEST1. The variants were found in exon 2. Bidirectional Sanger sequencing of parents further confirmed that both were heterozygous for the same variant (Fig. 4). Upon further inquiry, we ascertained a history of consanguinity.

Six months following TCP treatment, her vision improved to 20/250 in each eye with IOP between 14 and 16 mmHg in both eyes without anti-glaucoma medications. The ACD remained stable around 3.5 mm and there was no change in fundus examination with no recurrence of retinoschisis on OCT.

## Discussion and conclusions

Here we reported a novel autosomal–recessively inherited c.1 A > G variant in BEST1 associated not only with vitreoretinal interface abnormalities, but also high hyperopia and angle closure glaucoma. The pan-ocular abnormalities seen in BEST1-associated phenotypes may indicate an additional and important role of the RPE-specific bestrophin-1 protein in ocular development, potentially through interaction with the transcription factors orthodenticle homeobox 2, microphthalmia-associated transcription factor, and cone-rod homebox [6]. Although the exact mechanism for the pan-ocular abnormalities in ARB is still unknown, this case represents the first report of successful treatment of both ACG and retinoschisis in ARB with lens extraction and synechiolysis staged with low dose TCP.

Lens extraction has become first-line treatment in the management of angle closure in patients with or without co-existing cataract, as age-related lens growth plays a major role in the mechanism of ACG [7]. Lens extraction in ACG can relieve pupillary block, markedly decrease angle crowding, and improve the position of anteriorly displaced ciliary body processes, thus widening the angle [8]. In contrast, the 26-year-old patient described here did not demonstrate anterior chamber deepening following lens extraction, suggesting that the mechanism of angle closure in this case may not be pupillary block with an enlarged lens. More likely, pressure from the posterior segment led to anterior chamber shallowing by pushing the iridolenticular diaphragm forward. Although we cannot completely rule out malignant glaucoma after the lens extraction, her presentation was not typical due to a stable ACD without a vicious circle of flat anterior chamber. Low dose TCP resulted in gradually improved ACD combined with resolution of IOP elevation, likely by shrinking the ciliary body, inducing posterior ciliary body rotation [9], then straightening the lens zonules. TCP could also theoretically destroy zonules and disrupt the anterior hyaloid face, equalizing the pressure between anterior and posterior segments [10]. All of them lead to posterior displacement of the iridolenticular diaphragm, the same treatment mechanism for malignant glaucoma. But in consideration of ACD significant improvement since post-TCP day 4, there should be other explanations.

Quigley et al. [11] believed that poor vitreous flow conductivity contributed to the pathogenesis of ACG. They posited that choroidal expansion creates a high pressure environment, and poor vitreous flow conductivity prevents aqueous outflow, further increasing pressure in the posterior segment. As a result, the lens-iris diaphragm moved forward leading to angle closure. Our patient’s choroid was thicker than normal subjects, which would be consistent with this hypothesis. It could also explain why this patient’s symptoms got worse during pregnancy with water retention, which might aggravate choroidal expansion [12]. Histologically, the choroid, along with iris stroma and ciliary body, are all derived from the mesoderm. The iris thickening, ciliary body swelling, and thickened choroid seen in this patient may all relate to her underlying genetic change. Besides thickened choroid, her intact and unliquefied virteous played an important role in conducting the force from the thickened choroid. Anterior vitreous liquefaction, which occurs due to post-TCP inflammation, may decrease vitreous integrity, absorb the force from choroidal expansion, and increase flow conductivity through the vitreous cavity. Lowering of the peripheral vitreous pressure in the setting of liquefication could also restore the ciliary body orientation and deepen the anterior chamber. This explanation matches our patient’s clinical presentation where high IOP was not completely reversed until there was evidence of vitreous liquefaction.

In this patient, TCP led not only to resolution of IOP elevation and shallow anterior chamber, but also resolution of retinoschisis. Some have proposed that the retinoschisis in ARB is secondary to RPE dysfunction, but this would not explain improvement following TCP [1, 13]. Longitudinal vitreous traction may play a significant role in the pathogenesis of retinoschisis. Additionally, epiretinal membranes at the vitreoretinal interface, vitreoretinal condensations, and vitreous strands to the macula have all been reported in the spectrum of bestrophinopathy, which may represent some abnormal development of the vitreous in patients with ARB [13]. Vitreous liquefaction decreases vitreous traction to the macula and may resolve macular retinoschisis. The improved retinal structure following TCP provides insight in treating retinoschisis in patients with bestrophinopathy through inducing vitreous liquefication or addressing the vitreoretinal interface.

This case is unique not only in its description of a novel variant for ARB, but also in its identification of a successful treatment regimen for ACG and retinoschisis in ARB using low dose TCP. Compared to older ACG patients, patients with ARB are younger and have high posterior pressure due to choroidal thickening in combination with unliquefied vitreous. This pathophysiology may apply to other types of ACG in younger patients and influence treatment algorithms as well. Standard trabeculectomy may lead to a shallow or flat anterior chamber with post-operative malignant glaucoma due to posterior pressure from the vitreous. Previous studies have shown that younger ACG patients, especially with ARB, develop post-operative malignant glaucoma more frequently than older ACG patients after trabeculectomy [3, 14]. Surgeries which create additional outflow pathways, like trabeculectomy, increase the pressure gradient between the anterior and posterior segments. Solid and unliquefied vitreous in these younger patients blocks fluid flow and triggers misdirection of aqueous into or behind the vitreous body. Therefore, surgeries that affect the vitreous body flow conductivity, such as anterior vitrectomy to remove vitreous or cyclophotocoagulation to liquify vitreous, should be considered for patients with potential high posterior pressure, particularly those with a thick choroid or younger patients with solid vitreous.

This report is the first to identify a novel pathogenic ARB variant. Additionally, it improves our understanding of the role of the vitreous body and vitreous liquefication in the pathogenesis for patients with ACG and ARB. Though more research is needed, this case suggests that treatments that induce vitreous liquefaction could be used to treat patients with ARB or other kinds of ACG and to avoid significant post-operative complications.

## Data Availability

All data generated or analyzed during this study are included in this published article. If readers want to consult the original data, they can contact the corresponding author. We will provide it.

## Abbreviations

ARB: Autosomal recessive bestrophinopathy
RPE: Retinal pigment epithelium
ACG: Angle-closure glaucoma
IOP: Intraocular pressure
UBM: Ultrasound biomicroscopy
SD-OCT: Spectral domain optical coherence tomography
ACD: Anterior chamber depth
TCP: Transscleral cyclophotocoagulation
